# UXT oligomerization is essential for its role as an autophagy adaptor

**DOI:** 10.1016/j.isci.2025.112013

**Published:** 2025-02-13

**Authors:** Min Ji Yoon, Jugeon Park, MinHyeong Lee, Jiyeon Ohk, Tae Su Choi, Eun Jung Choi, Hosung Jung, Chungho Kim

**Affiliations:** 1 Department of Life Sciences, Korea University, Seoul 02841, Republic of Korea; 2 Department of Anatomy, Graduate School of Medical Science, Brain Korea 21 Project, Yonsei University College of Medicine, Seoul 03722, Republic of Korea; 3 Potomac Affinity Proteins, 11305 Dunleith Pl, North Potomac, MD 20878, USA

**Keywords:** Biochemistry, Molecular biology

## Abstract

SQSTM1/p62 serves as an autophagy receptor that binds to ubiquitinated misfolded proteins and delivers them to the phagophores for removal. This function can be augmented by autophagy adaptors, such as UXT. Here, by *in silico* structural homology modeling, we demonstrated that UXT can potentially form a hexameric structure to bind to misfolded proteins. Importantly, the UXT hexamer can assemble into a high-order oligomer via β hairpins positioned outside of each hexamer, facilitating the formation and efficient removal of protein aggregates. Consistently, the high-order oligomer of UXT was found to be essential for inducing the efficient clearance of SOD1(A4V) aggregates, in both *in vitro* and *in vivo*. Collectively, our research emphasizes the crucial importance of UXT oligomerization in its role as an autophagy adaptor and explains why the structurally and functionally similar prefoldin, which lacks such high-order oligomerization capacity, is employed for the refolding of individual misfolded proteins, but not autophagy.

## Highlights

• UXT can form a hexamer capable of binding to misfolded proteins
• UXT hexamers can assemble into a high-order oligomer via extra β hairpins
• The high-order oligomerization of UXT is crucial for removal of protein aggregates

## Graphical abstract

**Figure:**
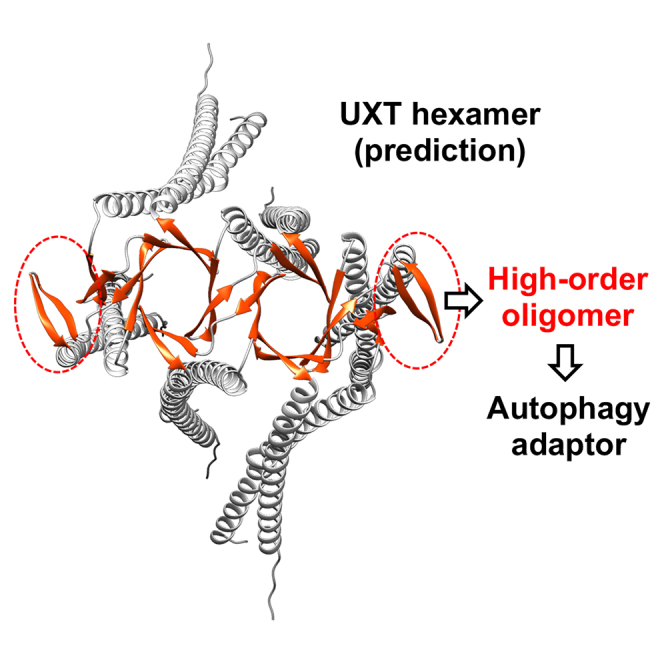

## Introduction

Autophagy is a catabolic process in which intracellular materials, including protein aggregates, pathogens, and intracellular organelles, are encircled by phagophores, delivered to lysosomes, and degraded by lysosomal enzymes.^1^ Specifically, the autophagy-mediated degradation of protein aggregates, termed aggrephagy, is responsible for preventing proteotoxicity resulting from misfolded protein aggregates, especially in neuronal cells.^2^ Several autophagy receptors that selectively bind to aggregates and deliver them to the LC3 on the autophagosome membrane during aggrephagy have been identified.^3^ SQSTM1/p62 (hereafter referred to as p62) is a receptor that contains a ubiquitin-associated domain for binding to protein aggregates and an LC3-interacting region for targeting the complex to the phagophore.^3^^,^^4^ Additionally, p62 contains an N-terminal PB1 domain that can polymerize in a head-to-tail manner, resulting in the formation of a linear p62 polymer^5^ and possibly increasing its avidity toward protein aggregates. The p62-protein aggregate complexes are observed in cells as sphere-shaped particles with a diameter of approximately 1 μm, known as p62 bodies,^4^ before they are finally targeted to protein degradation. Therefore, formation of p62 bodies is considered an essential step in p62-dependent autophagic removal of protein aggregates.^6^

The function of p62 as an autophagy receptor is facilitated by its interactors or autophagy adaptors, such as autophagy-linked FYVE protein (ALFY) and DAXX. For example, ALFY binds to p62^7^ and phosphatidylinositol 3-phosphate on the phagophore membrane through its FYVE domain,^8^ thereby promoting the accumulation of p62 bodies containing protein aggregates and autophagic machinery. DAXX is another p62 interactor that induces liquid-phase condensation of p62 by inducing p62 oligomerization,^9^ possibly by increasing the avidity of p62 for its target proteins. Recently, UXT, a prefoldin-like chaperone protein, was found to bind to p62 directly and increase p62 clustering for efficient targeting of protein aggregates.^10^ Therefore, these adaptors appear to amplify one (or more) of the three major intrinsic properties of the aggrephagy receptor p62—protein aggregate binding, phagophore targeting, or oligomerization—for efficient autophagic removal of protein aggregates.

Earlier studies have demonstrated other roles of UXT, as a co-activator of androgen receptor signaling^11^ and a transcriptional co-factor of nuclear factor-kappa B transcription.^12^ UXT is associated with γ-tubulin^13^ and aggregated mitochondria.^14^ In addition, based on the sequence similarity of UXT with the α subunit of prefoldin chaperone, UXT has been proposed to form a part of the prefoldin-like co-chaperone hexameric complex.^15^ These known functions of UXT appear to rely on its ability to form multi-protein complexes, such as protein aggregates, transcriptional enhancements, or chaperone complexes.

In this study, we aimed to elucidate the connection between the structural properties of UXT and its functions, particularly focusing on its role in autophagy. Because of its propensity to form large protein complexes, obtaining a purified, structurally defined unit of UXT is challenging. Consequently, we utilized the recently developed structural tool, AlphaFold,^16^ and revealed that UXT can form a hexamer that can bind to protein aggregates. We then validated these structural predictions and functional consequences using diverse biochemical and cell biology approaches and *in vivo* animal model systems. We believe our approach used here not only offers insights into the molecular mechanisms underlying UXT’s functions in targeting protein aggregates and influencing aggregate dynamics, but also serves as an exemplary demonstration how the rapidly advancing artificial intelligence tools can be leveraged in traditional life sciences research.

## Results

### Homology modeling of the structure of UXT predicts its targeting to protein aggregates

**Figure 1:**
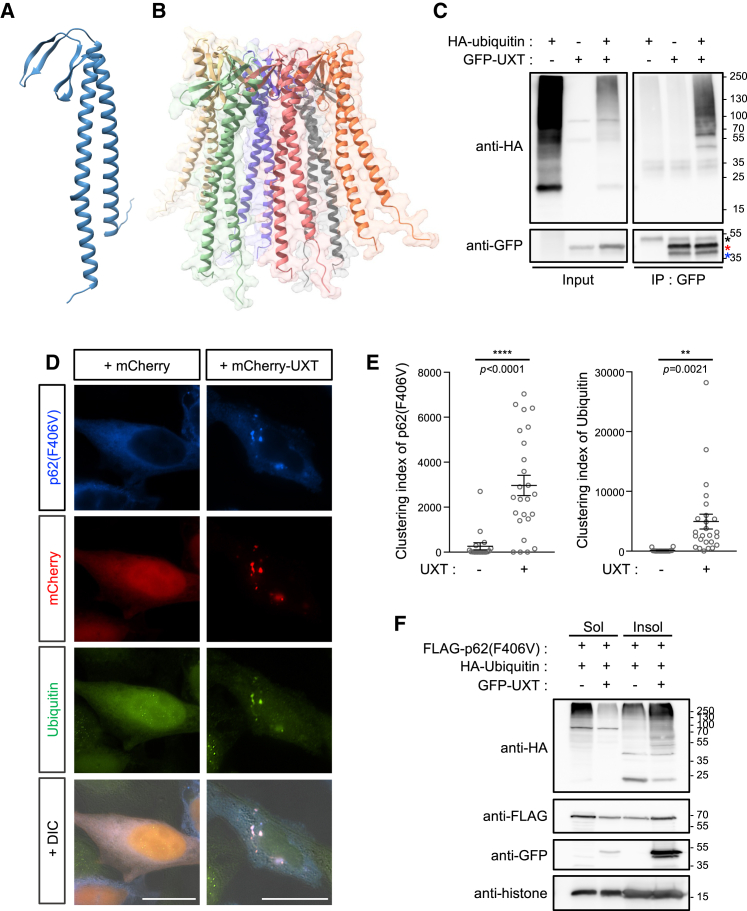
Homology modeling of the UXT structure predicts its targeting to protein aggregates (A) Structure of UXT predicted using AlphaFold. (B) Homology modeling prediction of the UXT oligomer using AlphaFold. Each color corresponds to a UXT monomer. (C) HEK293T cells were transfected with HA-ubiquitin and GFP-UXT, and their interaction was demonstrated by the detection of HA-ubiquitin in precipitates of GFP-UXT. The predicted position of GFP-UXT is marked with a red asterisk. Anti-GFP IgG bands and possibly degraded GFP-UXT bands are marked with black and blue asterisks, respectively. (D) p62KO/HeLa cells were transiently transfected with FLAG-p62(F406V) and mCherry-UXT or mCherry-only control. After 8 h of treatment with 2.5 μM MG132 and 50 nM bafilomycin-A1, cells were stained using anti-FLAG and anti-ubiquitin antibodies, followed by anti-mouse-Alexa Fluor 350 and anti-rabbit-Alexa Fluor 488 antibodies, respectively. Representative fluorescence images and their merged composites with DIC images are shown. Scale bar, 20 μm. (E) Clustering indexes of p62(F406V) and ubiquitinated proteins have been calculated and as described,^10^ and represented as scatterplots (*n* = 18 and 25 cells, respectively, examined over three independent experiments, mean ± SEM). ∗∗, *p* < 0.01; ∗∗∗∗, *p* < 0.0001 (two-tailed unpaired *t*-test). (F) HEK293T cells were transiently transfected with FLAG-p62(F406V), HA-ubiquitin, and GFP-UXT or vector, and then treated with 50 nM bafilomycin A1 and 2.5 μM MG132 for 12 h. Proteins in the Triton X-100-soluble or -insoluble fractions were detected using western blot.

Prefoldin consists of two prefoldin αs and four prefoldin βs, which form an α_2_β_4_ hexameric jellyfish-like structure to function as a molecular chaperone that delivers unfolded proteins to chaperonin.^17^^,^^18^ The two α-helices at the N- and C-terminal ends of each prefoldin subunit form the tentacles that bind misfolded proteins. Additionally, prefoldin α subunit contains two β hairpins, while prefoldin β subunit contains one β hairpin, collectively forming β-barrel structures crucial for the hexameric assembly.^19^^,^^20^ Similar to the prefoldin α subunit, UXT was predicted to have two α helices at both ends and two β hairpins in the middle^15^; our structural prediction also confirmed this configuration (Figures 1A and S1A; Data S1). Based on the similarity of UXT to prefoldin, and previous observations of its oligomerization potential,^10^^,^^13^ we tested whether UXT could be assembled into a homomeric hexamer. Our modeling study predicted that UXT indeed adopts a prefoldin hexamer-like structure (Figure 1B; Data S2). In this configuration, two UXT subunits behave just like prefoldin α, while four UXT subunits take the prefoldin β positions, but leave four additional β hairpins (Figure S1B, arrows). Interestingly, two out of the four hairpins make β strands with neighboring β-barrel structures (Figure S1B, gray arrows), while the other two hairpins are positioned outside the hexamer (Figure S1B, black arrows). Our model also predicted that the internal region, surrounded by a total of twelve α helices in the hexamer, forms a hydrophobic surface resembling that of the prefoldin hexamer (Figure S1C), suggesting its ability to interact with the hydrophobic surfaces of misfolded proteins.

Utilizing the structural information on UXT, we first tested its interaction with misfolded proteins. When green fluorescent protein (GFP)-tagged UXT and hemagglutinin (HA)-tagged ubiquitin were expressed in HEK293T cells and UXT was immunoprecipitated, we found that some misfolded proteins had co-precipitated with it (Figure 1C). If this is the case, we hypothesized that UXT is not only recruited indirectly to protein aggregates by interacting with the well-known aggregate-targeting autophagy receptor p62,^10^ but may also be recruited directly to protein aggregates and, conversely, play a role in recruiting p62. To test this hypothesis, we introduced a mutation in the ubiquitin-binding domain of p62, F406V, which impairs the interaction between p62 and the ubiquitinated misfolded proteins.^21^^,^^22^ Upon introducing the p62(F406V) mutant into p62 knock-out HeLa cells (HeLa/p62KO), we observed a lack of induction of p62 body formation, with the mutant dispersed throughout the cytoplasm (Figure 1D). However, co-transfection with UXT resulted in the accumulation of ubiquitinated proteins in UXT-positive regions (Figure 1D), leading to a substantial increase in p62 body formation and aggregate clustering (Figure 1E). Consistent with this finding, the presence of UXT decreased the amount of ubiquitinated proteins and p62 mutant in the soluble fraction (Figure 1F, lanes 1 and 2), concurrently elevating the localization of both the p62 mutant and the ubiquitinated proteins in the detergent-insoluble fraction (Figure 1F, lanes 3 and 4). Taken together, these results suggested that the UXT oligomer can bind to protein aggregates, possibly through its hydrophobic surface, and recruit p62, independent of the ability of p62 to bind to ubiquitin. However, we note that UXT appears to exhibit some target specificity, as suggested by the differing band patterns between input and UXT-immunoprecipitated samples (Figure 1C). Consistently, UXT associates with the aggregation-prone SOD1(A4V)^23^^,^^24^ in our previous study,^10^ but not with another aggregation-prone protein, ataxin-1(92Q)^25^ (Figure S2) possibly due to differences in cellular localization.

### UXT enhances the stability of protein aggregation

**Figure 2:**
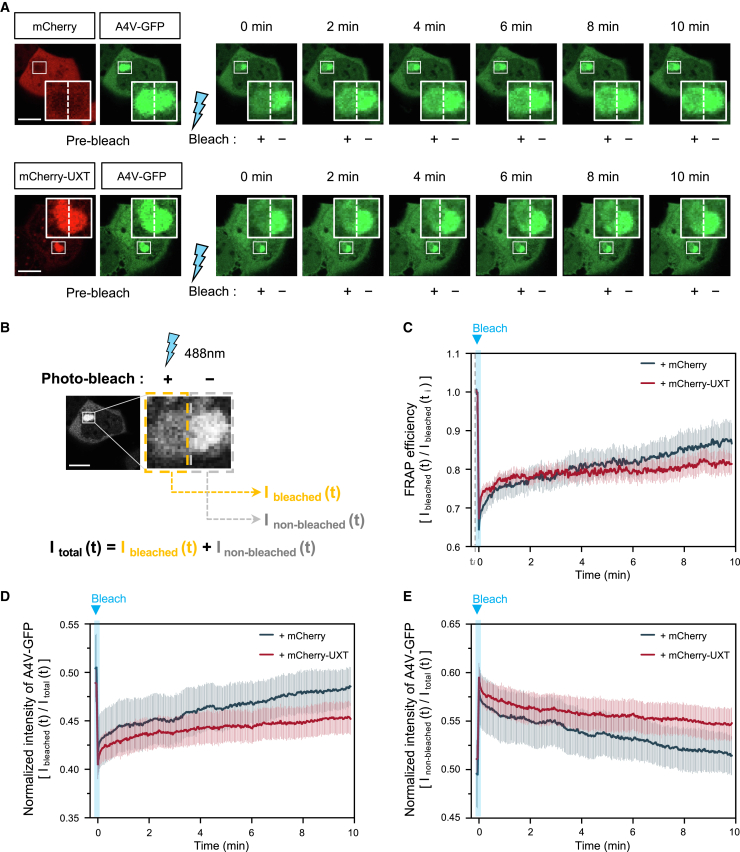
UXT regulates the dynamics of protein aggregation (A) HEK293T cells were transfected with SOD1(A4V)-GFP along with either mCherry control vector or mCherry-UXT. After 24 h of incubation, the cells were treated with 2.5 μM MG132 and 50 nM bafilomycin-A1 for 8 h. Photobleaching was performed using a 488-nm laser on half regions of large aggregates, and the signal recovery or diffusion in the bleached or non-bleached region was observed for 10 min, at intervals of 2 s. The representative images are displayed at 2 min intervals. Scale bar, 10 μm. (B) Experimental scheme. (C and D) FRAP analysis of SOD1(A4V)-GFP in the bleached region. The mean intensity in the bleached region was normalized against that at the initial time (t_*i*_) in (C) or against that in the total region in (D). (E) FRAP analysis of SOD1(A4V)-GFP in the non-bleached region. The mean intensity in the non-bleached region was normalized against that in the total region. mCherry vector-transfected cells (blue, *n* = 6); mCherry-UXT WT-transfected cells (red, *n* = 8). The results are presented as mean ± SEM.

The interaction between UXT and misfolded proteins was detected as a punctate staining pattern (Figure 1D), which prompted the hypothesis that UXT promotes the formation of compact and stable protein aggregates. To investigate this hypothesis, we used SOD1(A4V) mutant fused with GFP and conducted a fluorescence recovery after photo bleaching (FRAP) assay to compare the dynamics of the SOD1(A4V)-GFP aggregates in the presence or absence of UXT. To eliminate the potential effect of proteolysis on this process, treatments with MG132 and Baf-A1 were carried out to inhibit the ubiquitin-proteasome system and autophagy, respectively. Half of the SOD1(A4V)-GFP aggregates co-transfected with either mCherry or mCherry-UXT were subjected to bleaching using a 488-nm laser, while the remaining half served as a control (Figure 2A). Fluorescence intensities of the bleached and non-bleached regions were measured for 10 min post-bleaching, at intervals of 2 s (Figure 2A), to calculate I_bleached_ (t) and I_non-bleached_ (t) at each time point (t), in min (Figure 2B). When FRAP efficiency was calculated by normalizing I_bleached_ (t) against the intensity before bleaching, it was evident that co-expression of UXT resulted in decreased FRAP efficiency compared to that of the control vector (Figure 2C), indicating decreased mobility of aggregates in the presence of UXT. In addition, when I_bleached_ (t) and I_non-bleached_ (t) were normalized against the total fluorescence intensities of the aggregate region [I_total_ (t)], calculated in terms of I_bleached_ (t) + I_non-bleached_ (t), there was an increase in the normalized I_bleached_ (t) (Figure 2D), while the normalized I_non-bleached_ (t) showed a decrease (Figure 2E), indicating movement of SOD1(A4V)-GFP within the aggregates. Again, the degree of movement, or change in relative fluorescence in both regions, was diminished in the presence of UXT (Figures 2D and 2E, red lines), as compared to that in the control (Figures 2D and 2E, blue lines). Together, these results suggested that UXT contributes to the stabilization of SOD1(A4V) aggregates, potentially facilitating a more efficient autophagic clearance.

### UXT forms oligomers

**Figure 3:**
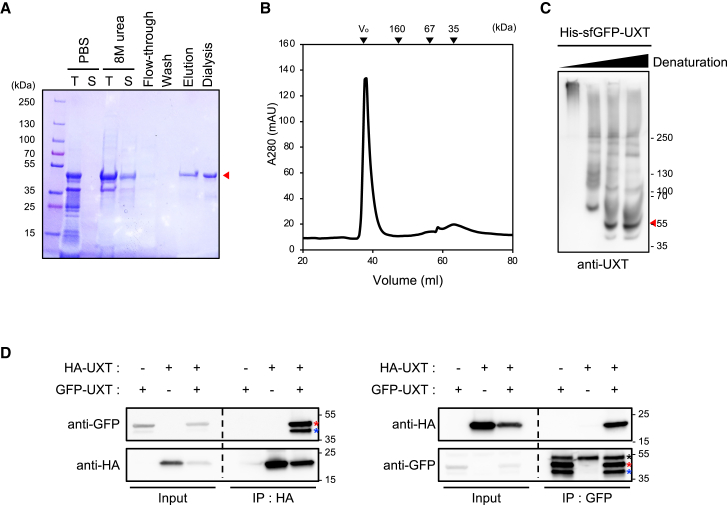
UXT forms oligomers (A) 6×His-sfGFP-UXT, overexpressed in BL21(DE3) cells, was purified under denaturation conditions by means of binding of Ni-Sepharose beads, followed by a refolding step. T, total cell lysates; S, soluble fraction. The red arrowhead indicates the target band. (B) The oligomeric state of recombinant 6×His-sfGFP-UXT was analyzed using size-exclusion chromatography. (C) Samples prepared in (A) were analyzed using native polyacrylamide gel electrophoresis, followed by a western blot with a serial gradient of SDS concentrations for denaturation and an anti-UXT antibody. Native gel analysis of the recombinant 6×His-sfGFP-UXT with application of a serial gradient of SDS concentrations revealed UXT signals near the well and close to the 250 kDa marker, while the predicted size of 6×His-sfGFP-UXT monomer has been indicated with a red arrowhead. (D) HEK293T cells were transfected with HA-UXT and GFP-UXT, and the presence of GFP-UXT in precipitates of HA-UXT or vice versa was detected. The predicted position of GFP-UXT is marked with a red asterisk. Anti-GFP IgG bands and possibly degraded GFP-UXT bands are marked with black and blue asterisks, respectively.

Since successful oligomerization of UXT appears to be essential for its function in interacting with protein aggregate by forming a hydrophobic core (Figures S1B and S1C), we aimed to validate the ability of UXT to form oligomers. To facilitate the purification of the UXT protein in *E. coli*, we fused a 6×His tag and superfolder GFP^26^ to UXT. After purification using Ni-Sepharose beads under denaturing conditions followed by refolding (Figure 3A) owing to its notable insolubility, UXT was subjected to size-exclusion chromatography (SEC). UXT eluted near the void volume of the column, significantly exceeding the expected molecular weight of 46.9 kDa (Figure 3B). Consistent with the results of the SEC, the purified UXT exhibited a high molecular weight upon native polyacrylamide gel electrophoresis (PAGE) (Figure 3C, lane 1). In contrast, exposure of UXT to an elevated concentration of sodium dodecyl sulfate (SDS) converted it into a monomer (Figure 3C, red arrow). Moreover, co-immunoprecipitation assays of HA- and GFP-tagged UXT tags expressed in HEK293T cells demonstrated reciprocal interactions (Figure 3D). These results indicated the potential of UXT to form oligomers.

Our model indicates that the oligomerization of UXT is propelled by β hairpins forming two β barrels, with α helices contributing to intra-subunit coil-coiled structures, but not inter-subunit interactions (Figure 1B). To confirm this, we serially deleted N- and C-terminal regions in α helices (Figures S3A and S3B) and investigated their contribution to UXT oligomerization. Among these deletion mutants, UXT(ΔN′C′), which harbors intact β-hairpin regions with the most of hydrophobic portions in the α helices deleted, was expressed in a soluble form in HEK293T cells (Figure S3C, red box). This deletion mutant tagged with 6*×*His was purified from *E. coli* in a soluble form (Figure S3D) and analyzed using native PAGE (Figure S3E). Similar to the wild-type (WT) UXT (Figure 3C), the α helix-deleted mutant was observed as high-molecular weight bands upon native PAGE (Figure S3E, black arrowheads), but a monomer-sized band under denaturing conditions (Figure S3E, red arrowhead). Purification of the same deletion mutant using glutathione S-transferase (GST) fusion followed by GST cleavage (Figure S3F) showed essentially the same oligomerization trends as those observed upon native PAGE (Figure S3G). Taken together, these findings indicated that UXT forms homo-oligomers, and the α-helix regions of UXT are non-essential for its oligomerization process.

### The extra β hairpins of UXT are essential for forming a high-order oligomer

**Figure 4:**
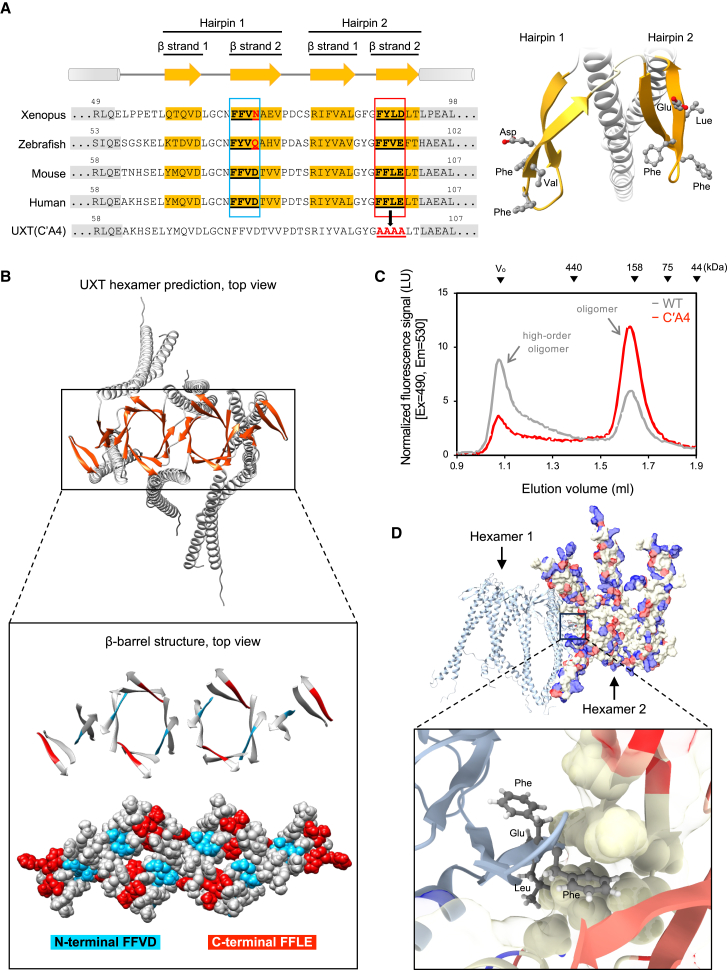
Extra β hairpins of UXT are essential for forming a high-order oligomer (A) Secondary structure diagram, with the amino acids adjacent to the β strands of UXT in vertebrates shown (left). The amino acids within the β hairpin and α helix motifs are highlighted in yellow and gray shading, respectively. The FFXD/E motifs in UXT are underlined. The amino acid residues in FFLE motif in human UXT are substituted with alanines in the UXT(C'A4) mutant. The structural model specifically highlighting the FFXD/E motifs within the β strands of human UXT is shown (right). (B) Structural model of the UXT hexamer predicted using AlphaFold. The α helices and β strands are colored in gray and orange, respectively (upper). The β strands are shown in an enlarged image and the regions of the specific N- or C- terminal FFXD/E amino acids, FFVD and FFLE, are colored blue and red, respectively (lower). (C) The detergent-free cell lysates of HEK293T cells expressing GFP-UXT WT or GFP-UXT(C′A4) were prepared, and their components were separated using SEC with a fluorometer. Gray, UXT WT; red, UXT(C′A4). (D) The UXT dodecamer was modeled using AlphaFold. Each hexameric UXT is depicted with either a ribbon or surface structure. In the hexameric structure, the FFLE extends toward the hydrophobic surface of the adjacent hexamer. In the surface representation of hexamer 2, the electrostatic potential is visualized on the molecular surface. Negatively and positively charged amino acids are shown in red and blue, respectively.

Based on the molecular mass of hexameric prefoldin determined by means of gel filtration,^27^ ∼200 kDa, we expected the molecular mass of hexameric UXT to be similar. However, in most cases, the size of purified UXT in the SEC and native PAGE was much larger than expected (Figures 3B and 3C). As our UXT hexamer modeling suggests the presence of additional β hairpins, which are not integral to maintaining the hexamer structure, but rather positioned outside of the hexamer (Figure S1B, black arrows), we hypothesize that these β hairpins may play a role in establishing additional interactions between the hexamers, which may account for the bigger size observed in the SEC. Interestingly, we observed that two β hairpins in UXT have distinct amino acid sequences of the FFXD/E motif (F, phenylalanine; X, an amino acid with a hydrophobic residue) (Figure 4A), which is also found in the amyloid beta region, possibly mediating its fibril formation.^28^ In the N-terminal β hairpin, FFVD was observed, while in the C-terminal β hairpin, FFLE was observed. Especially, the second FFXD/E motif is relatively highly evolutionarily conserved (Figure 4A) and also localized in the β hairpin outside of the hexamer (Figure 4B). To investigate the importance of this unique region in inter-hexameric interactions, alanine substitutions were made in the second FFXD/E motif on the C-terminal β hairpins, to generate the UXT(C′A4) mutant. The mutant was fused to GFP and expressed in HEK293T cells, following which the detergent-free lysate of the cells was analyzed using an analytical SEC system equipped with a fluorometer. In this assay, WT UXT displayed two major peaks (Figure 4C). The first peak was observed near the void volume, corresponding to the high-order oligomer. The second peak, whose presence seemed to depend on the cellular context, was generally observed at ∼200 kDa, potentially corresponding to the UXT hexamer.^10^ However, in the C′A4 mutant, only the second peak of ∼200 kDa, but not the peak of the high-order oligomer of either monomer, was evident in the SEC analysis (Figure 4C), suggesting that the C-terminal FFXD/E motif is involved in the inter-hexameric interactions. In addition, when analyzed by native PAGE, purified UXT(ΔN′C′) with the C′A4 mutation (Figure S4A) did not show a band that likely corresponds to the high-order oligomer seen in the UXT(ΔN′C′) without the mutation (Figure S4B). Consistent with these results, when two UXT hexamers were assembled *in silico* using AlphaFold, the hydrophobic side chains of the FFLE sequences in these hexamers extended toward each other’s hydrophobic surfaces and established hydrophobic interactions (Figure 4D; Data S3), supporting our experimental data.

### High-order oligomerization of UXT is essential for its aggregate-clearance function

**Figure 5:**
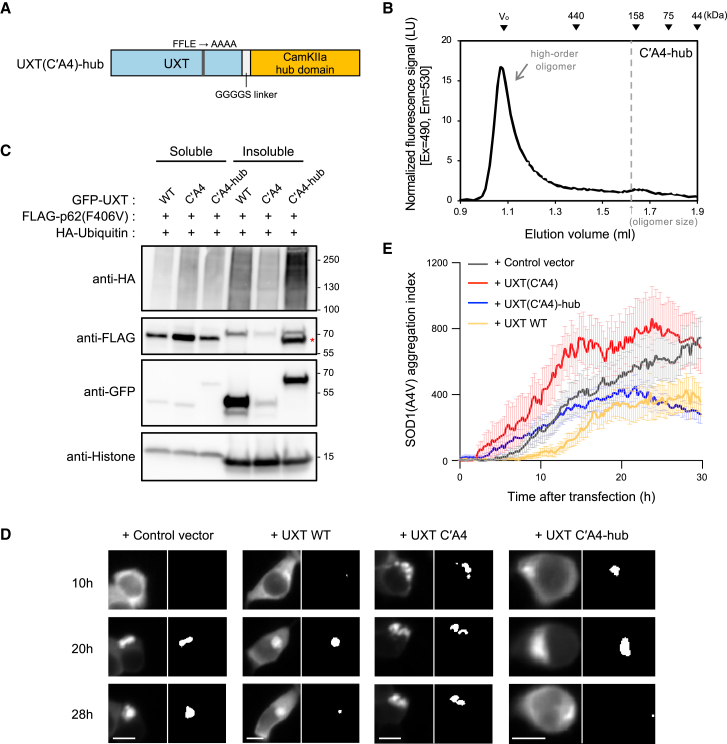
High-order oligomerization of UXT is essential for its aggregate-clearance function (A) Schematic of the UXT-hub chimeric mutant. (B) The detergent-free cell lysate of HEK293T cells expressing GFP-UXT(C′A4)-hub was prepared and its components were separated using SEC with a fluorometer. (C) HEK293T cells were transiently transfected with FLAG-p62(F406V), HA-ubiquitin with GFP-UXT WT or mutants, and then subjected to MG132 and Baf-A1 treatment. The expression of each protein in the Triton X-100-insoluble or -soluble lysates was detected using western blot. The red asterisk indicates the GFP-UXT(C′A4)-hub signal left from previous anti-GFP blot. (D) HEK293T cells were transiently co-transfected with SOD1(A4V)-GFP and either mCherry-UXT WT or mutants, or a control consisting only of mCherry. Following a 1-h transfection, the cells were treated with 1 μM MG132. Fluorescence images of SOD1(A4V)-GFP were then captured for a total of 30 h, at intervals of 15 min. Representative images at 10, 20, and 28 h are shown, along with black and white images indicating the aggregate regions identified using a MATLAB code.^10^ Scale bar, 10 μm. (E) The aggregation indexes were calculated using an in-house MATLAB code^10^ and averaged for different groups. Vector control- (gray, *n* = 19), mCherry-UXT WT- (yellow, *n* = 23), mCherry-UXT C′A4 (red, *n* = 12), and mCherry-UXT C′A4-hub-transfected cells (blue, *n* = 20). The results are presented as mean ± SEM.

Next, we investigated the importance of high-order oligomerization of UXT for its functions. For the specific evaluation of high-order oligomerization, we artificially engineered oligomerization of UXT(C′A4) by adding the hub domain of Ca^2+^/calmodulin-dependent protein kinase II, known to form dodecameric or tetradecameric oligomers.^29^ The resulting protein, UXT(C′A4)-hub (Figure 5A), was largely eluted near the void volume and to a minimal extent at the size of a hexamer in the fluorescence-based analytical SEC system, indicating that it forms high-order oligomers more efficiently than wild type (Figure 5B).

After characterizing the UXT variants with or without the capacity to form high-order oligomers, we employed them in assays established to elucidate the functional roles of UXT. The UXT-mediated increase in p62 body formation of the p62(F406V) mutant and aggregate clustering (Figures 1D and S5A, left panels) was substantially reduced by the high-order oligomerization-defective UXT(C′A4) mutant (Figure S5A, middle panels), but was restored by UXT(C′A4)-hub to the levels comparable to those of wild type UXT (Figure S5A, right panels). Similarly, UXT’s ability to target p62(F406V) and misfolded proteins to the detergent-insoluble fraction (Figures 1F and 5C, lane 4) was diminished by UXT(C′A4) (Figure 5C, lane 5) but recovered with UXT(C′A4)-hub (Figure 5C, lane 6). When we tested the effect of UXT C′A4 mutant on the stabilization of SOD1(A4V) aggregates using FRAP, however, we observed no significant difference compared to wild-type UXT (Figure S5B). This suggests that, although the C′A4 mutation reduces high-order oligomer formation, the mutant may still form partial oligomers upon targeting to protein aggregates, retaining some stabilizing effect. Finally, the C′A4 mutant also showed reduced LC3 targeting, which was restored by the C′A4-hub (Figure S5C). As previously described,^10^ the targeting abilities of UXT to protein aggregates and LC3 are related to the autophagy-dependent clearance of protein aggregates. To examine the role of high-order UXT oligomer in aggregate clearance, we assessed SOD1(A4V)-GFP aggregate levels in HEK293T cells transiently transfected with UXT variants (Figure S6). However, in this transient transfection system, SOD1(A4V) levels are influenced by both the expression and degradation timing of SOD1(A4V) and UXT, making reliable measurement of clearance challenging. To address this limitation, we employed time-lapse imaging to monitor SOD1(A4V) aggregate clearance in cells with comparable SOD1(A4V) and UXT expression levels at the time of analysis. Indeed, upon examination by means of live-cell imaging, SOD1(A4V)-GFP expressed in HEK293T cells formed aggregates that persisted throughout the experimental time frame, whereas the growing aggregates were eventually cleared in cells co-transfected with UXT (Figures 5D and 5E; Video S1). In this analysis as well, the aggregate-clearance function of UXT was reduced by the C′A4 mutation, but again restored effectively by UXT(C′A4)-hub (Figures 5D and 5E; Video S1). These results indicated that the high-order oligomer of UXT is essential for its aggregate-clearance function.

Video S1. Time-lapse image of SOD1(A4V) aggregates formation, related to Figure 5

### High-order oligomerization of UXT is essential for its protective function against proteotoxicity

**Figure 6:**
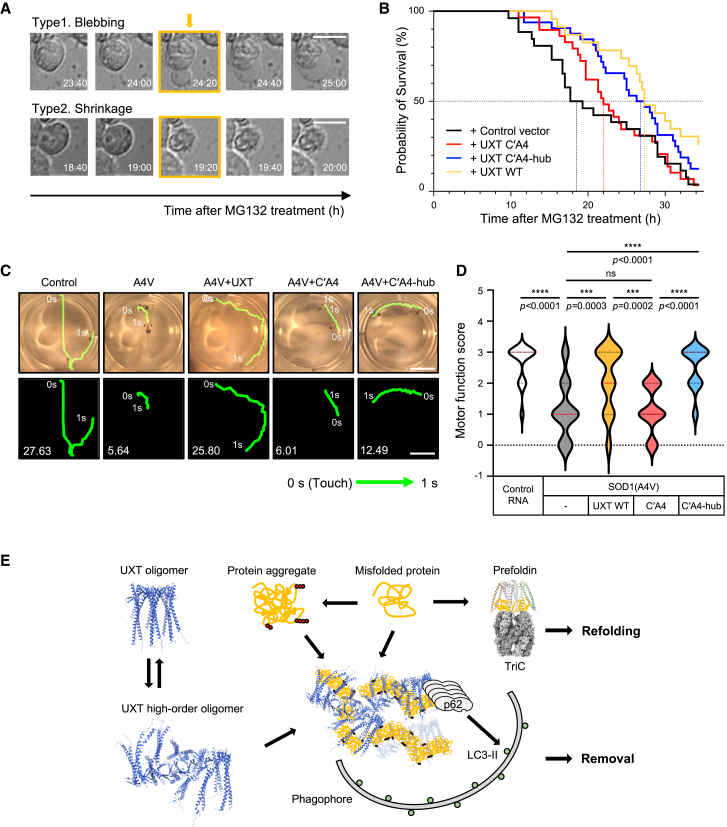
The high-order oligomerization of UXT is essential for its protective function against proteotoxicity (A) Cellular death markers, blebbing or shrinkage, in HEK293T cells. Time after MG132 treatment (h) is indicated in the right corner of each image. Scale bar, 20 μm. (B) Survival analysis of HEK293T cells transfected with GFP-SOD1(A4V), along with mCherry control vector (black), mCherry-UXT WT (yellow), mCherry-UXT(C′A4) (red), or mCherry-UXT(C′A4)-hub (blue), upon MG132 treatment. (C) Evaluation of motor function in tadpoles expressing SOD1(A4V) and UXT WT or mutants (C′A4 or C′A4-hub) in the central nervous system, through targeted microinjection. Motor function was assessed in terms of touch-induced swimming responses. Each tadpole was touched on its tail at least three times, and the representative experiment is shown. The trace represents the swimming trajectory within 1 s, while the number in the left corner indicates the distance (mm) covered in 1 s. Scale bar, 5 mm. (D) Scoring of the tadpole’s motor function was carried out based on the velocity and swimming distance after touch. The scoring system ranged from 0 to 3, with the following criteria: 0, no response at all, but normal cardiac muscle; 1, no swimming, but moving tail; 2, reduced response, swimming distance <1/4^th^ diameter of the well; 3, normal response, swimming distance >1/3^rd^ diameter of the well. Control RNA, *n* = 28; SOD1(A4V), *n* = 45; SOD1(A4V) with UXT WT, *n* = 38; SOD1(A4V) with UXT(C’A4), *n* = 40; SOD1(A4V) with UXT(C’A4)-hub, *n* = 29, Dunn’s multiple comparison *p*-values adjusted with the Bonferroni method. ns, *p* > 0.5; ∗∗∗, *p* < 0.001; ∗∗∗∗, *p* < 0.0001. (E) Schematic representation of the possible mechanism for the role of UXT as an autophagy adaptor and comparison of its function with that of prefoldin in the misfolded protein control process. TriC, PDB ID: 7WU7.

Prolonged presence of protein aggregates arising from the accumulation of misfolded proteins exceeding the capacity of the ubiquitin-dependent proteasome system results in proteotoxicity and triggers apoptotic cell death.^30^^,^^31^ To experimentally mimic this proteotoxic condition, we expressed SOD1(A4V) in HEK293T cells in the presence of the proteasome inhibitor MG132. This condition caused two types of cell death in SOD1(A4V)-transfected cells—blebbing or shrinkage (Figure 6A; Video S2). Using these markers, we monitored the progression of cell death in HEK293T cells co-transfected with GFP-fused SOD1(A4V) and mCherry-fused UXT variants over time using a fluorescence microscope equipped with temperature and humidity controls. Cells expressing WT UXT exhibited resistance to proteotoxicity, as compared to the control, with 50% cell death observed at approximately 27 h and 18 h after MG132 treatment, respectively (Figure 6B, yellow vs. black lines). However, this protective effect was diminished in cells expressing the UXT(C′A4) mutant, resulting in 50% cell death at approximately 22 h (Figure 6B, red). The diminished protective effect was restored by introducing the potential for formation of a high-order oligomer, as seen in the case of UXT(C′A4)-hub (Figure 6B, blue), where 50% cell death was observed at approximately 26 h. These results suggested that the formation of a high-order oligomer by UXT may play a protective role against cell death induced by protein aggregates.

Video S2. Two types of cell death in SOD1(A4V)-transfected cells – blebbing or shrinkage, related to Figure 6

Finally, to validate the significance of high-order oligomerization of UXT *in vivo*, we injected RNA encoding GFP-tagged SOD1(A4V) into dorsal blastomeres, the precursors of neuronal cells, and monitored the motor neuron function of the subsequent tadpoles, utilizing this approach as an amyotrophic lateral sclerosis model.^24^ The swimming response of the tadpoles was observed under a microscope equipped with a video camera (Figure 6C). Tadpoles expressing SOD1(A4V) exhibited significant defects in their swimming response, such as reduced responsiveness to touch or an inability to swim, indicating impaired motor function, as compared to the control tadpoles (Figures 6C and 6D; Video S3). Remarkably, the introduction of UXT WT or UXT(C′A4)-hub in *Xenopus* markedly alleviated the loss of motor function induced by SOD1(A4V) (Figures 6C and 6D; Video S3), possibly by eliminating the SOD1(A4V) aggregate in the motor neurons.^10^ In contrast, UXT(C′A4), incapable of forming a high-order oligomer, did not exhibit significant protective effects (Figures 6C and 6D; Video S3). In conclusion, our findings demonstrated that the high-order oligomer formation by UXT alleviates protein aggregate-induced proteotoxicity.

Video S3. Loss of motor function in SOD1(A4V)-expressing tadpoles and the effect of UXT WT or mutants introduction, related to Figure 6

## Discussion

In this study, we performed a structural analysis of UXT using a recently developed structure modeling tool, AlphaFold, based on the experimentally determined structure of its homologous protein prefoldin. The structural studies first revealed that just like prefoldin, UXT can make a homomeric hexamer by establishing a β-barrel structure in the middle through two β hairpins from each UXT subunit, with two α helices from each subunit forming a 12 tentacle-like structure that can bind to a misfolded protein possibly through its hydrophobic surface (Figure 1B). This prediction was verified by means of biochemical and cell biology studies, which showed that UXT can bind to ubiquitinated proteins (Figures 1C and 1D) and promote their aggregation (Figure 2). The second structural aspect of the predicted UXT hexamer was found to be the presence of additional β hairpins outside of the hexameric structure. We found that the β hairpins, particularly the FFXD/E motif within the region, may play a role in the inter-hexameric interactions of UXT, leading to the formation of a high-order oligomer of UXT (Figures 3 and 4). Furthermore, our structural model, which depicts the interaction between two hexameric UXT units, also illustrates that the centers of the tentacles from each hexamer face different directions, creating an angle of approximately 90° between them (Figure 4D). Consequently, once the high-order oligomer is established, the resulting complex presents misfolded protein-binding sites in various directions, which allows for the effective binding and aggregation of misfolded proteins, by entangling with them (Figure 6E).

We propose that this distinctive structural feature of UXT is the primary characteristic that differentiates the function of UXT from that of the structurally similar prefoldin. The absence of additional β hairpins in the prefoldin hexamer (Figure S1B) prevented further oligomerization of the hexamers. The resulting prefoldin with one hydrophobic core is more efficient at binding to an individual misfolded protein, and may be employed to deliver misfolded proteins individually into the chaperonin complex, for refolding.^32^^,^^33^ In contrast, UXT, with its multiple hydrophobic cores, can bind to multiple misfolded proteins, making it more efficient at forming protein aggregates and delivering them to the autophagy machinery (Figure 6E). Thus, it appears that these structurally and functionally similar prefoldins and UXT play two similar but distinct roles in determining the fate of misfolded proteins: refolding or destruction. This also implies that the precursor of UXT, initially functioning as a chaperone, may have evolved to acquire the potential for oligomerization, thereby becoming integrated into the autophagy system.

In this study, we demonstrated that the ability to form a high-order oligomer induces the rapid removal of SOD1(A4V) aggregates in HEK293T cells (Figure 5D) and enhances protective functions against SOD1(A4V) aggregates in motor neurons (Figure 6D), both of which are autophagy-dependent processes, as suggested by the evidence that the inhibition of autophagy by means of bafilomycin treatment or deletion of the autophagy receptor p62 impedes the functions of UXT.^10^ How is the high-order oligomerization potential of UXT associated with increased autophagy? Endogenously expressed UXT has a short lifespan and undergoes rapid degradation via the ubiquitin-dependent proteasomal system.^10^^,^^34^ However, in the presence of misfolded proteins, we envision that UXT can bind to the misfolded proteins and be stabilized as a hexamer, after which the misfolded protein-loaded UXT hexamers form high-order oligomers, facilitating the aggregation of misfolded proteins within the limited space of the oligomer. Once the protein aggregates are formed, numerous UXT molecules in the complex expose p62-binding sites, thereby enhancing the avidity of the aggregates toward p62. We believe that the resulting increased and efficient attachment of the autophagy receptor p62 to the complex can promote its delivery to phagophores and autophagic removal for protecting against proteotoxicity. Since the protective effects were also observed with C′A4-hub, which likely forms a differently shaped high-order oligomer, it appears that the increased avidity of UXT resulting from oligomerization, rather than the overall structure of the high-order oligomer, is the main factor influencing its effects in our assays.

In conclusion, the present study provides a better understanding of the molecular mechanisms underlying the functions of UXT in targeting protein aggregates and influencing aggregate dynamics. It also establishes that the ability of UXT to target protein aggregates and protect against proteotoxicity depends on its ability to form high-order oligomers. Based on these results, we suggest that oligomerization-dependent control of UXT function can be utilized as a therapeutic strategy against neurodegenerative diseases and other protein aggregation-related disorders.

### Limitations of the study

Due to the challenge of obtaining a purified, structurally defined unit of UXT forming large protein complexes, our structural analyses relied on computational modeling. While these analyses were validated by various conventional cell biological and biochemical techniques, obtaining structural data on the UXT high-order oligomer formation through cryo-electron microscopy and/or X-ray crystallography would provide a clear picture of the precise mechanism by which UXT gathers protein aggregates in high-order oligomers.

## Resource availability

### Lead contact

Further information and requests for resources and reagents should be directed to and will be fulfilled by the corresponding author, Professor Chungho Kim (chungho@korea.ac.kr).

### Materials availability

All reagents and antibodies were obtained from commercial sources, as documented in the key resources table. Plasmids generated in this study are available from the lead contact upon request.

### Data and code availability

• Data: all study data are included in the article and/or supplemental information.
• Code: this paper does not report previously unpublished custom code. An analytical code used for analysis is available at https://zenodo.org/records/4563694 and is stated in the key resources table.
• Additional information: any additional information required to reanalyze the data reported in this paper is available from the lead contact upon request.

## Acknowledgments

This work was supported by the Basic Science Research Program (2022R1A2B5B02001854, RS-2023-00221182, and NRF-2022R1C1C2008329) and a 10.13039/501100002642Korea University Grant.

## Author contributions

M.J.Y. and C.K. designed the study. M.J.Y. (biochemistry and cell biology); J.P., J.O. (*Xenopus* experiment); M.L. (biochemistry); T.S.C. and E.J.C. (structural biology) performed the experiments. M.J.Y., T.S.C., E.J.C., H.J., and C.K. analyzed the data. M.J.Y. and C.K. wrote the manuscript, which was edited by T.S.C., E.J.C., and H.J.

## Declaration of interests

The authors declare no competing interests.

## STAR★Methods

### Key resources table

**Table:** 

REAGENT or RESOURCE	SOURCE	IDENTIFIER
**Antibodies**		

Mouse monoclonal anti-Ubiquitin antibody	abcam	Cat# ab7254; RRID: AB_305802
Rabbit monoclonal anti-Ubiquitin antibody	abcam	Cat# ab134953; RRID: AB_2801561
Mouse monoclonal anti-p62 antibody	abcam	Cat# ab56416; RRID: AB_945626
Rabbit monoclonal anti-p62 antibody	abcam	Cat# ab109012; RRID: AB_2810880
Mouse polyclonal anti-UXT antibody	abcam	Cat# ab168678
Rabbit monoclonal anti-LC3A/B antibody	Cell Signaling Technology	Cat# 12741; RRID: AB_2617131
Rabbit monoclonal anti-Histone H3 antibody	Cell Signaling Technology	Cat# 4499
Mouse monoclonal anti-GFP antibody	Santa Cruz	Cat#sc-9996; RRID: AB_627695
Mouse monoclonal anti-FLAG antibody	Sigma	Cat#F1804; RRID: AB_262044
Rabbit polyclonal anti-HA antibody	Thermo Fisher	Cat#71-5500; RRID: AB_2533988
Anti-rabbit IgG-Alexa Fluor 488 antibody	Thermo Fisher	Cat#A32731; RRID: AB_143165
Anti-mouse IgG-Alexa Fluor 350 antibody	Thermo Fisher	Cat#A11068; RRID: AB_2534112
Goat Anti-Mouse-HRP Conjugated	Thermo Fisher	Cat# 31430; RRID: AB_228307
Goat Anti-rabbit-HRP Conjugated	Thermo Fisher	Cat# 31460; RRID: AB_228341

**Bacterial and virus strains**		

*Escherichia coli* strain DH5α	Enzynomics	CP011
*Escherichia coli* strain BL21(DE3)	New England Biolabs	C3040I
*Escherichia coli* strain Stbl3	Enzynomics	CP111
Lentivirus	This study	

**Chemicals, peptides, and recombinant proteins**		

DMEM/Dulbecco’s Modified Eagles Medium	Hyclone	SH30243.01
Fetal Bovine Serum	Gibco	16000-044
Penicillin Streptomycin	Hyclone	SV30010
Trypsin-EDTA	Thermo Fisher	SH30042.01
isopropyl β-D-1-thiogalactopyranosid	Sigma	I6758
Ni Sepharose™ High Performance	Cytiva	7-5268-0
Protease Inhibitor Cocktail	Roche	11836170001
Triton X-100	Sigma-Aldrich	T9284
Lipofectamine™ 3000	Invitrogen	L3000150
Polyethylenimine, linear	Sigma	408727
Fluorescence Mounting Medium	Agilent	S3023
Hoechst Nucleic Acid Stains	Invitrogen	H3569
Imidazole	Sigma	I2399
Urea	Sigma	U1250
BSA	USB	10857
Superdex200 increase 5/150 GL	Cytiva	28990945
MG132	Sigma	M7449
Bafilomycin A1	Sigma	B1793
Dimethyl Sulfoxide (DMSO)	Simga	472301
protein A agarose	ThermoFisher	20334
protein G agarose	ThermoFisher	20399

**Critical commercial assays**		

mMESSAGE mMACHINE® Kit	Invitrogen	AM1344

**Experimental models: Cell lines**		

Human: HEK293T	ATCC	
Human: HeLa	ATCC	
Human: HeLa/p62KO	Yoon et al.^10^	

**Experimental models: Organisms/strains**		

*Xenopus tropicalis*	Xenopus 1 Corp. (MI, USA).	

**Oligonucleotides**		

Forward primer for p62(F406V) mutagenesis 5′-atgctgtccatgggcgtgtctgatgaaggcggc-3′	BIONICS	Oligonucleotide Synthesis
Reverse primer for p62(F406V) mutagenesis 5′-gccgccttcatcagacacgcccatggacagcat-3′	BIONICS	Oligonucleotide Synthesis
Forward primer for UXT(C’A4) mutagenesis 5′-gtggccctgggatatggtgccg ccgctgcattgacactggcagaagct-3′	BIONICS	Oligonucleotide Synthesis
Reverse primer for UXT(C’A4) mutagenesis 5′-agcttctgccagtgtcaatgcagcgg cggcaccatatcccagggccac-3′	BIONICS	Oligonucleotide Synthesis
Forward primer for UXT subcloning to pRSF vector 5′-cggaattcgcgacgccccctaag-3′	BIONICS	Oligonucleotide Synthesis
Reverse primer for UXT subcloning to pRSF vector 5′-ccgctcgagtcaatggtgaggctt-3′	BIONICS	Oligonucleotide Synthesis
Forward primer for UXT(C’A4)-hub cloning 5′-atctcgagggaggggggggatcccggaaacaggaaatt-3′	BIONICS	Oligonucleotide Synthesis
Reverse primer for UXT(C’A4)-hub cloning 5′-tagggccctcagtggggcaggacggaggg-3′	BIONICS	Oligonucleotide Synthesis

**Recombinant DNA**		

pcDNA3-HA-ubiquitin	Prof. Eui-Ju Choi from Korea University	
pcDNA3-HA-p62	Yoon et al.^10^	
pcDNA3.1-FLAG-p62(F406V)	This paper	
pEGFP-n3-SOD1(A4V)	Yoon et al.^10^	
pcDNA3-HA-UXT	Yoon et al.^10^	
pcDNA3-HA-UXT(C’A4)	This paper	
pcDNA3-HA-UXT(C’A4)-hub	This paper	
pmCherry-c1-UXT	Yoon et al.^10^	
pmCherry-c1-UXT(C’A4)	This paper	
pmCherry-c1-UXT(C’A4)-hub	This paper	
pEGFP-c1-UXT	Yoon et al.^10^	
pEGFP-c1-UXT(C’A4)	This paper	
pEGFP-c1-UXT(C’A4)-hub	This paper	
pRSF-6×His-sfGFP-UXT	This paper	
pUC57-CamKII-hub	BIONICS	Gene Synthesis

**Software and algorithms**		

MATLAB R2023aSoftware	The MathWorks Inc.	https://www.mathworks.com/products/matlab.html; RRID: SCR_001622
ImageJ	National Institutes of Health	https://imagej.nih.gov/ij/
Prism10.0.3	Graphpad Software	https://www.graphpad.com
ZEN microscopy software (ZEISS)	ZEISS	https://www.zeiss.com/
NIS-Elements AR (Nikon)	Nikon	RRID: SCR_014329
AlphaFold	Jumper et al.^16^	https://github.com/deepmind/alphafold
UCSF Chimera	Resource for Biocomputing, Visualization, and Informatics (RBVI), UCSF	www.rbvi.ucsf.edu/chimera/
Custom code	Yoon et al.^10^	https://doi.org/10.5281/zenodo.4563694

**Other**		

Xenbase	Zahn et al.^34^	http://www.xenbase.org/
Fluorescence microscope	Nikon	Eclipse Ti
Confocal microscope	Carl Zeiss	LSM900
Bio imaging analyzer	GE Healthcare Life Sciences	LAS 4000 mini

### Experimental model and study participant details

#### Animal model

Wild-type adult *Xenopus tropicalis* were purchased from Xenopus1 (Dexter, MI). Approximately 10 adult male and female frogs were housed separately in a 40-L tank containing dechlorinated tap water maintained at 24°C. *Xenopus tropicalis* embryos were generated by means of *in vitro* fertilization and raised in 0.1× Modified Barth’s saline at 21°C–24°C. *Xenopus* were staged based on the tables provided by Nieuwkoop and Faber (1967) and Xenbase (http://www.xenbase.org/).^35^ All experimental procedures adhered to protocols approved by the Institutional Animal Care and Use Committee of Yonsei University College of Medicine under IACUC-2023-0250.

#### Cell lines

HEK293T (ATCC) and HeLa/p62KO cells^10^ were cultured in Dulbecco’s modified Eagle’s medium (HyClone) supplemented with 10% (v/v) fetal bovine serum (Gibco) and 1% penicillin-streptomycin (HyClone), at 37°C, in an incubator containing 5% CO2. To authenticate HeLa/p62KO cells, we frequently performed immunoblotting using anti-p62 antibody.

### Method details

#### Plasmids

The pcDNA3-HA-ubiquitin construct was provided by Prof. Eui-Ju Choi from Korea University. Constructs including pcDNA3-HA-p62, pEGFP-n3-SOD1(A4V), pmCherry-c1-UXT, pEGFP-c1-UXT, and pcDNA3-HA-UXT, previously described,^10^ were used to generate pcDNA3.1-FLAG-p62 and various UXT deletion mutants. Mutant constructs, pcDNA3.1-FLAG-p62(F406V) and UXT(C′A4), were generated by PCR-based mutagenesis. The hub domain of Ca^2+^/calmodulin-dependent protein kinase II, synthesized by BIONICS Co., Ltd, was used to create UXT(C′A4)-hub mutant construct via restriction enzyme digestion. PCR-amplified UXT or its deletion mutants were cloned into bacteria expression vectors to generate pRSF-6×His-sfGFP-UXT, pET28a-UXT(ΔN′C′)-6×His, and pGEX-4T-1-UXT(ΔN′C′).

#### Protein sample preparation

The recombinant proteins were expressed in *E. coli* BL21(DE3) cells. Cells were grown in Luria Bertani broth at 37°C, with agitation at 200 rpm, until the OD_600_ reached a value of 0.5. Subsequently, recombinant protein expression was induced using a final concentration of 0.2 mM isopropyl β-D-1-thiogalactopyranoside in the culture, following which the cells were cultured for 16 h at 18°C. The *E. coli* cell pellets were then resuspended in buffer A (20 mM Tris pH 8.0, 150 mM NaCl, and 2 mM DTT) and lysed by means of sonication. After centrifugation at 20,000  × *g* for 30 min, the resulting supernatant was transferred to Ni-Sepharose beads (Cytiva) packed in a column. The Ni-Sepharose beads were washed with buffer B (20 mM Tris pH 8.0, 150 mM NaCl, 2 mM DTT, and 10 mM imidazole) and eluted using a linear gradient of imidazole (0–500 mM). To purify insoluble proteins under denaturation conditions, after the initial centrifugation, the pellets were lysed in buffer C (20 mM Tris pH 8.0, 150 mM NaCl, and 8 M urea) and centrifuged at 20,000 × *g* for 30 min. The supernatant was applied to Ni-Sepharose beads packed into a column. The Ni-Sepharose beads were washed with buffer D (20 mM Tris pH 8.0, 150 mM NaCl, 8 M urea, and 10 mM imidazole) and eluted with 500 mM imidazole. The eluates were subsequently dialyzed sequentially with a urea gradient (from 6 to 0 M) in 20 mM Tris (pH 8.0), 150 mM NaCl, and 2 mM DTT, for 16 h.

#### Detergent-soluble and -insoluble fractionation

Cells were lysed with lysis buffer A [20 mM Tris pH 7.5, 150 mM NaCl, 1% Triton X-100, 1 mM EDTA, and protease inhibitor cocktail (Roche)], and 5% of the resulting lysate was set aside as total protein. Following centrifugation at 17,000 × *g* for 30 min at 4°C, the resulting supernatant was retained as the detergent-soluble fraction. The detergent-insoluble pellet was thoroughly washed with lysis buffer A and then solubilized in lysis buffer B (20 mM Tris pH 7.5, 150 mM NaCl, 1% Triton X-100, 1% SDS, 1 mM EDTA, and protease inhibitor cocktail). Immunoblotting was conducted with 15–20 μg of protein. Specific antibodies and their corresponding dilution factors are detailed in the ‘antibodies’ section.

#### Immunocytochemistry

One day before transfection, the cells were seeded on gelatin-coated coverslips. Plasmid constructs were transfected into cells on the coverslips using Lipofectamine 3000 (Invitrogen) and the cells were then incubated for 24 h. Following incubation, the cells were fixed with 3.7% formaldehyde and permeabilized using phosphate-buffered saline containing 0.1% Triton X-100 (PBST). Subsequently, the cells were treated with a blocking solution (1% bovine serum albumin, 22.52 mg/mL glycine, and 0.1% gelatin in PBST) and stained with the respective primary antibodies, which were diluted in the blocking solution. This was followed by staining with fluorophore-conjugated secondary antibodies diluted in the blocking solution. The antibodies used, along with their specific dilution factors, are detailed in the ‘Antibodies’ section. Finally, the coverslips were mounted on microscope slides using fluorescence mounting medium (Dako) with or without Hoechst (Invitrogen). Images were acquired using a fluorescence microscope (Ti-E, Nikon) equipped with a camera device (DS-Qi2, Nikon), and processed using NIS-Elements AR (Nikon) and/or ImageJ (NIH) software. The aggregation index was calculated using an analysis code^10^ available at https://zenodo.org/records/4563694.

#### FRAP analysis

One day before transfection, cells were seeded onto a 35-mm glass-bottom confocal dish (SPL Life Sciences). Plasmid constructs were then transfected into the cells using polyethylenimine and the cells were incubated for 24 h. FRAP measurements were conducted using an LSM 900 confocal microscope (Carl Zeiss). A 488-nm laser with 30% power was employed to bleach aggregates, and time-series images were acquired for 10 min, at intervals of 2 s. Confocal images of three frames before bleaching were captured, and the average fluorescence intensity in the test region served as the intensity of the initial time (t_*i*_), denoted as I_bleached_ (t_*i*_). Image processing was performed using the ZEN microscopy software (ZEISS), and ImageJ software (NIH) was used for further image processing and quantification of the FRAP measurements.

#### Gel filtration assay

UXT-induced oligomer formation was assessed using a SEC column equipped with a fluorometer. HEK293T cells expressing GFP-tagged UXT WT or mutants were resuspended in detergent-free buffer A (20 mM Tris pH 7.5, 150 mM NaCl, and 10% glycerol) and lysed by means of sonication. Following centrifugation at 20,000 × *g* for 1 h at 4°C, the resulting supernatants were combined with buffer B (20 mM Tris pH 7.5, 350 mM NaCl, and 0.4% C12E8) at a ratio of 1:1. After filtering the solution through a 0.2-μm filter, 10 μL of the solution was loaded onto a gel-filtration column that had been pre-equilibrated with buffer B, and the resulting elution profile was monitored by detecting the GFP signal (excitation at 490 nm and emission at 530 nm).

#### *Xenopus* experiments

To transcribe capped RNAs *in vitro*, mMESSAGE mMACHINE kit (Ambion) was used. Microinjection involved the introduction of a total of 450 pg of RNA per blastomere, comprising 200 pg encoding enhanced green fluorescent protein (EGFP)-fused SOD1 mutants or control (EGFP), 200 pg encoding UXT or control (mCherry), and 50 pg encoding a tracer (mCherry) into the two dorsal blastomeres, the precursor of the central nervous system. Each well in a 24-well culture plate housed one injected embryo. Each tadpole’s tail was touched at least three times using the tip of a plastic microloader, and their responses were observed under a dissection microscope equipped with a video camera.

#### Antibodies

The antibodies used for immunoblotting (IB), immunocytochemistry (ICC), immunoprecipitation (IP), and immunohistochemistry (IHC) are listed: mouse monoclonal anti-GFP [1:2500 (IB) and 1 μg (IP); sc-9996] from Santa Cruz Biotechnology; mouse monoclonal anti-FLAG [1:2500 (IB); F1804] and rabbit anti-histone H3 [1:5000 (IB); H0164] from Sigma; mouse [1:200 (ICC); ab7254] and rabbit [1:200 (ICC); ab134953] monoclonal anti-ubiquitin from Abcam; rabbit polyclonal anti-HA [1:2000 (IB) and 1 μg (IP); 71–5500], anti-rabbit [1:1000 (ICC); A11046] and anti-mouse [1:1000 (ICC); A11068] Alexa Fluor 350-conjugated IgG, and goat anti-mouse [1:10000 (IB); 31430] and anti-rabbit [1:10000 (IB); 31460] horseradish peroxidase-conjugated IgG from Thermo Fisher; anti-rabbit [1:1000 (ICC); 711-025-152] and anti-mouse [1:1000 (ICC); 715-025-151] TRITC-conjugated IgG, goat anti-rabbit [1:1000 (ICC); 711-096-152] and anti-mouse [1:1000 (ICC); 715-096-150] FITC-conjugated IgG from Jackson Laboratory.

### Quantification and statistical analysis

Statistical analyses were performed using Prism (version 10.0.3; GraphPad Software). Statistical parameters such as *p*-values, definitions of error bars with a measure of the center, and the specific statistical tests employed for analysis are provided in the Figure or Figure legends. Unless specified otherwise, each experiment was performed at least three times.

## Supplemental information

Document S1. Figures S1–S6

Data S1. Structural model of UXT monomer, related to Figure 5

Data S2. Structural model of UXT hexamer, related to Figure 5

Data S3. Structural model of UXT dodecamer, related to Figure 5
