# Supplementary material for: UXT oligomerization is essential for its role as an autophagy adaptor
Source: iScience. 2025 Feb 13;28(3):112013. doi: 10.1016/j.isci.2025.112013 (PMC11910115; doi:10.1016/j.isci.2025.112013)
Supplement: Document S1. Figures S1–S6 [file mmc1.pdf]

iScience, Volume *28*

Supplemental information

UXT oligomerization is essential for its role as an autophagy adaptor

Min Ji Yoon, Jugeon Park, MinHyeong Lee, Jiyeon Ohk, Tae Su Choi, Eun Jung Choi, Hosung Jung, and Chungho Kim

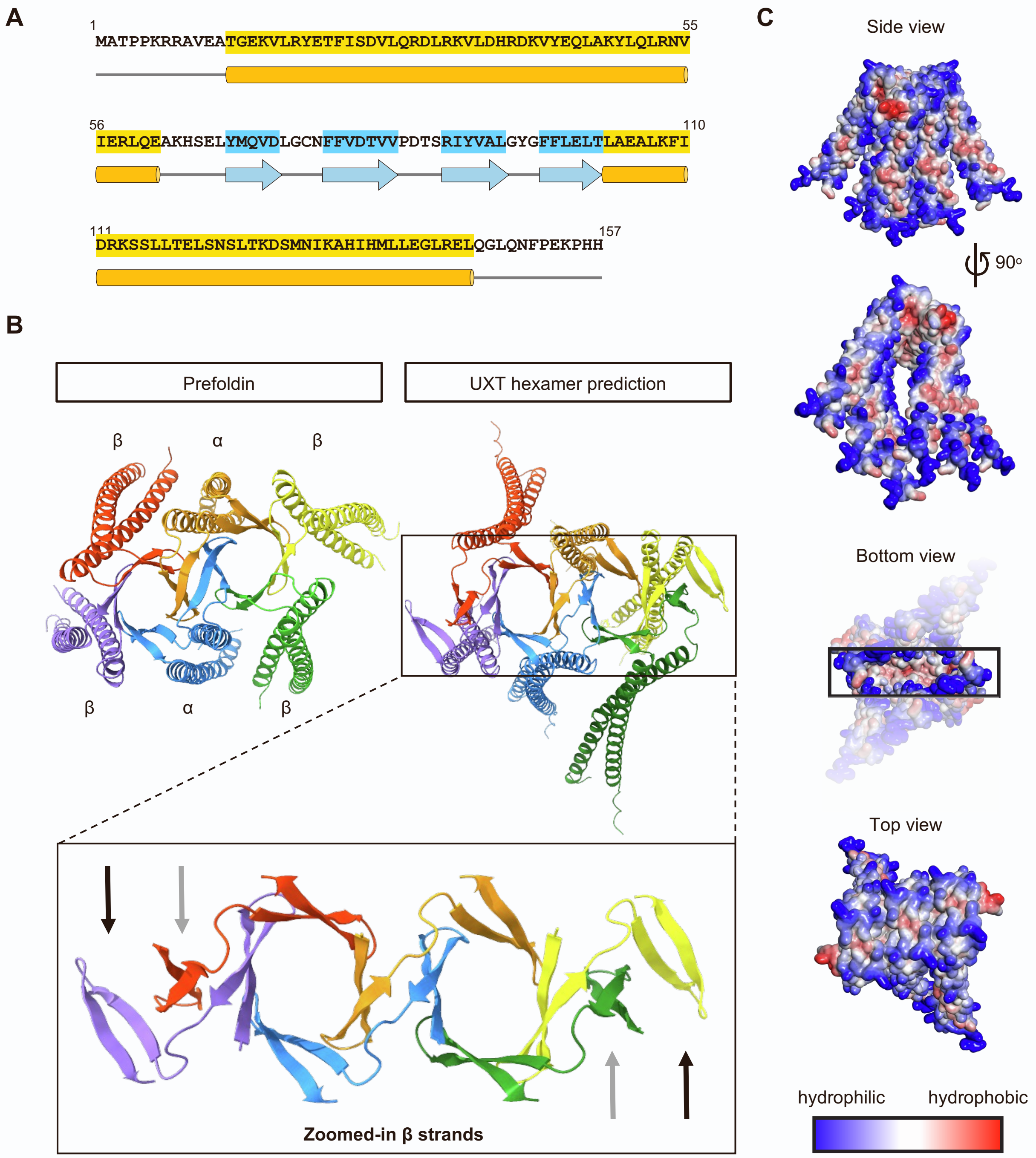

Figure S1. Prediction of the structures of UXT and UXT oligomer, related to Figure 1

(A) The amino acid sequence of UXT is displayed, with the secondary structure depicted below. Yellow shading represents amino acids involved in α helices, while sky blue shading represents those involved in β strands. (B) Comparison of the prefoldin structure and UXT hexamer structure predicted using AlphaFold homology modeling. In the prefoldin structure, each subunit (α or β subunit) is distinguished by a different color. In the predicted UXT hexameric structure, the monomeric UXT is distinguished by a different color. Gray arrows highlight the zoomed-in β hairpins of UXT in the prefoldin β positions, which contribute to the formation of β-barrel structures. Black arrows point to the two remaining hairpins positioned outside the hexamer. (C) In the predicted hexameric form of UXT, the anticipated internal region forming a hydrophobic surface is shown.

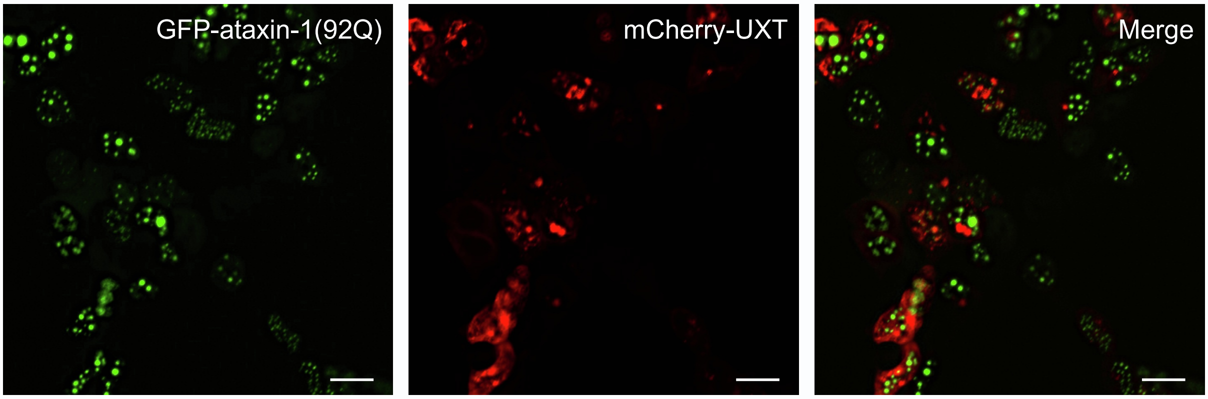

Figure S2. Different localization of UXT and ataxin-1(92Q) aggregates, related to Figure 1
HEK293T cells were co-transfected with GFP-ataxin-1(92Q) and mCherry-UXT. After 24 h, the cells were examined using fluorescence microscope. Scale bar, 20 μm.

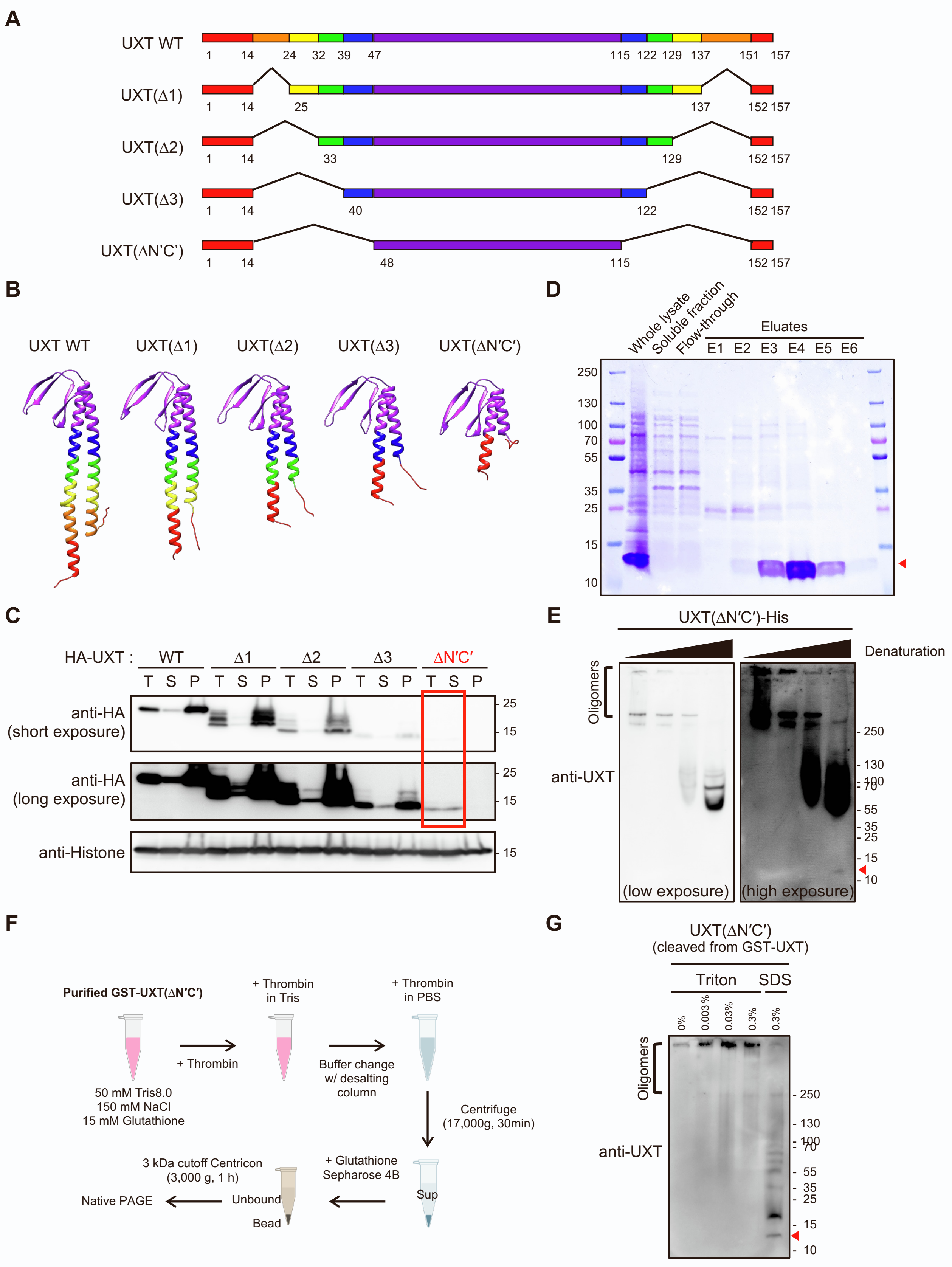

A
UXT WT
1 14 24 32 39 47 115 122 129 137 151 157
UXT(Δ1)
1 14 25 137 152 157
UXT(Δ2)
1 14 33 129 152 157
UXT(Δ3)
1 14 40 122 152 157
UXT(ΔN′C′)
1 14 48 115 152 157
B
UXT WT
UXT(Δ1)
UXT(Δ2)
UXT(Δ3)
UXT(ΔN′C′)
C
HA-UXT :
WT
Δ1
Δ2
Δ3
ΔN′C′
T S P
anti-HA (short exposure)
anti-HA (long exposure)
anti-Histone
25
15
D
Whole lysate
Soluble fraction
Flow-through
Eluates
E1 E2 E3 E4 E5 E6
250
130
100
70
55
35
25
15
10
E
UXT(ΔN′C′)-His
Denaturation
Oligomers
anti-UXT
(low exposure)
(high exposure)
250
130
100
70
55
35
25
15
10
F
Purified GST-UXT(ΔN′C′)
50 mM Tris8.0
150 mM NaCl
15 mM Glutathione
+ Thrombin
+ Thrombin in Tris
Buffer change w/ desalting column
+ Thrombin in PBS
Centrifuge (17,000g, 30min)
Sup
+ Glutathione Sepharose 4B
Unbound
Bead
3 kDa cutoff Centricon (3,000 g, 1 h)
Native PAGE
G
UXT(ΔN′C′)
(cleaved from GST-UXT)
Triton
SDS
0%
0.003 %
0.03%
0.3%
0.3%
Oligomers
anti-UXT
250
130
100
70
55
35
25
15
10

Figure S3. UXT forms oligomers independently of its α helices, related to Figure 3

(A) Diagram depicting the designs of various UXT(ΔN′C′) mutants with deletions in hydrophobic α helices at both the N- and C-terminals. (B) Structures of the UXT(ΔN′C′) mutants predicted using RoseTTAFold, where each color (red, orange, yellow, green, blue, and purple) corresponds to the respective region in (A). (C) Solubility test of the various UXT(ΔN′C′) mutants. SDS-PAGE analysis of whole-cell lysates (T), soluble fraction (S), and pellets lysed in SDS buffer (P), of cells expressing HA-tagged UXT WT or mutants. Expression in each fraction was determined using an HA antibody, with histone serving as a control. (D) UXT(ΔN′C′)-6×His protein expressed in BL21(DE3) cells was purified using Ni-Sepharose beads. The target protein band has been marked with a red arrowhead. (E) Samples prepared in (D) were analyzed using native PAGE, followed by western blot with a gradient of SDS concentrations for denaturation and an anti-UXT antibody. The UXT oligomer signals near the well and close to the 250 kDa marker are indicated with a bracket, while the signal for a monomeric UXT between 10~15 kDa are marked with a red arrowhead. (F) Outline of the purification process for GST-UXT(ΔN′C′). Following the purification of GST-UXT(ΔN′C′), the GST tag was removed using thrombin. (G) Native PAGE analysis of the recombinant UXT(ΔN′C′) using sequential Triton or SDS application. The predicted size of the UXT(ΔN′C′) monomer is indicated by a red arrowhead.

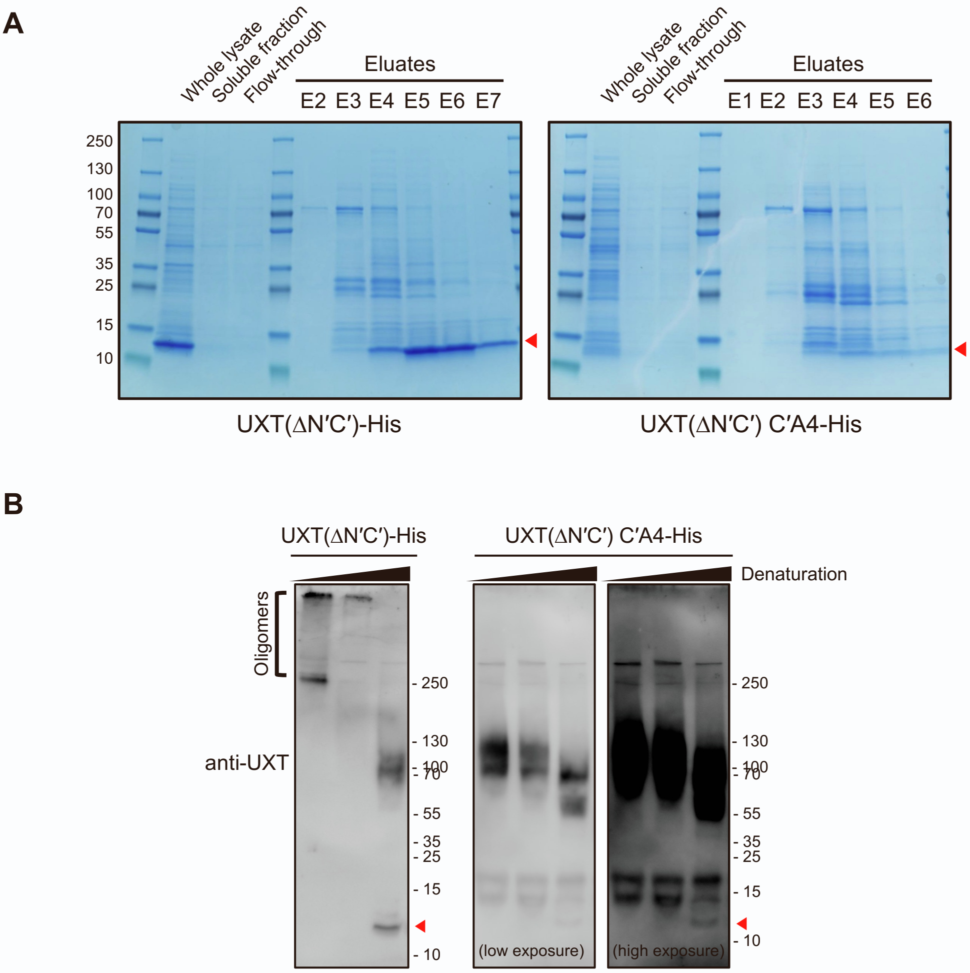

Figure S4. Effect of C′A4 mutation on the formation of high-order UXT oligomer, related to Figure 4
(A) UXT(ΔN′C′) or UXT(ΔN′C′) bearing C′A4 mutation was purified as in Figure S3D. The target protein bands are marked with red arrowheads. (B) Native PAGE analysis was conducted on the purified samples from (A), following the protocol outlined in Figure 3.

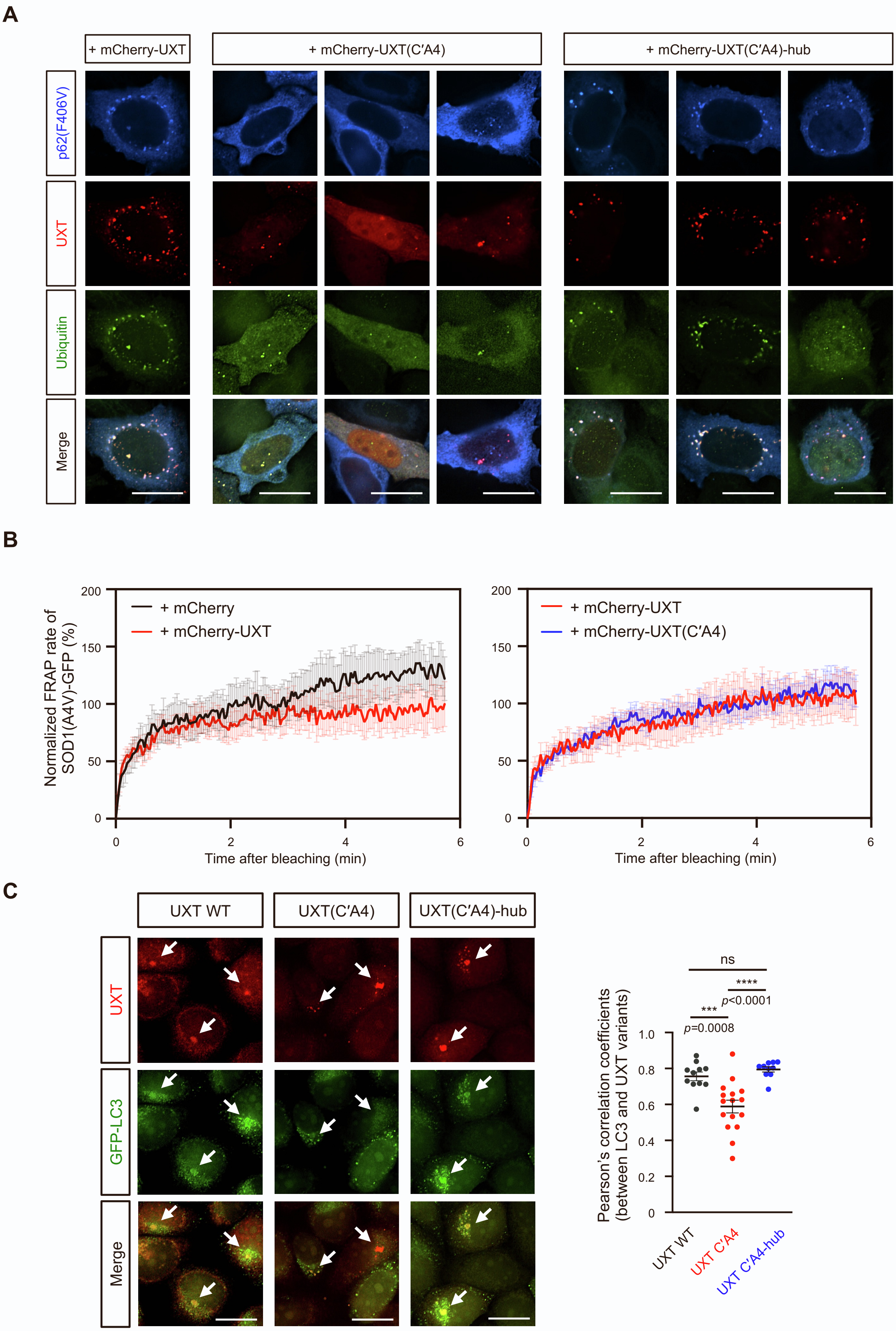
A
+ mCherry-UXT
+ mCherry-UXT(C′A4)
+ mCherry-UXT(C′A4)-hub
p62(F406V)
UXT
Ubiquitin
Merge
B
Normalized FRAP rate of SOD1(A4V)-GFP (%)
+ mCherry
+ mCherry-UXT
+ mCherry-UXT(C′A4)
Time after bleaching (min)
C
UXT WT
UXT(C′A4)
UXT(C′A4)-hub
UXT
GFP-LC3
Merge
Pearson's correlation coefficients (between LC3 and UXT variants)
ns
****
p<0.0001
***
p=0.0008
UXT WT
UXT C′A4
UXT C′A4-hub

Figure S5. The C′A4 mutation inhibits UXT binding to p62 and LC3, related to Figure 5

(A) HeLa/p62KO cells were transiently transfected with FLAG-p62(F406V) and either mCherry-UXT WT, mCherry-UXT(C′A4), or mCherry-UXT(C′A4)-hub, and analyzed as in Figure 1A. Scale bar, 20 μm. (B) HEK293T cells were co-transfected with SOD1(A4V)-GFP and either mCherry-UXT WT or C′A4 mutant. Fluorescence Recovery After Photobleaching (FRAP) analysis on those cells were performed as in Figure 2. The normalized FRAP rate was calculated by $I_{bleached}(t) - I_{bleached}(t=0) / I_{bleached,\ mCherry\text{-}UXT}(t=6) – I_{bleached,\ mCherry\text{-}UXT}(t=0)$ for each condition. (n=7 and 8 experiments for UXT WT and C′A4). The results from Figure 2 were normalized using the same method and displayed on the left side for comparison. Mean ± SEM is indicated. (C) HeLa cells stably expressing GFP-LC3 and mCherry-UXT variants were treated with 2.5 μM MG132 and 50 nM bafilomycin-A1 for 8 h, and analyzed by fluorescence microscope. Scale bar, 20 μm. Pearson’s correlation coefficients between LC3 and UXT variants were shown as scatter plots (n=11, 16, and 10 cells for UXT WT, C′A4, and C′A4-hub, respectively, mean ± SEM, one-way ANOVA using Tukey’s multiple comparison test.)

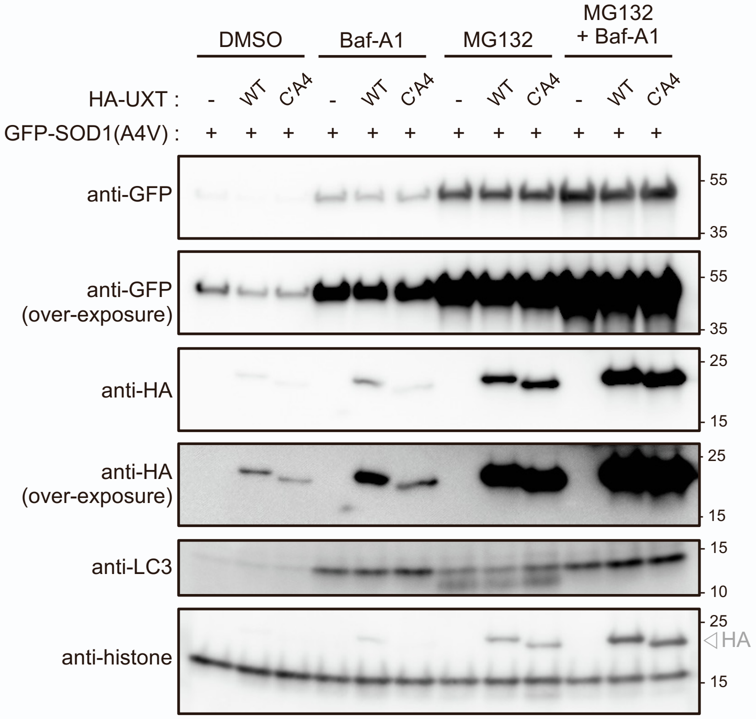

Figure S6. UXT mediated-clearance of SOD1(A4V) aggregates, related to Figure 5

SOD1(A4V)-GFP was co-transfected to HEK293T cells together with empty vector, HA-UXT wild type (WT) or HA-UXT(C′A4) mutant. At 24 h after transfection, cells were treated with 2.5 μM MG132 and 50 nM bafilomycin-A1 for 8 h, and then lysed with 1% triton X-100. The detergent-insoluble fractions were analyzed by western blots.
